# Supplementary material for: Multiregional representations of intertemporal decision making in human single neurons
Source: Sci Rep. 2025 Jul 8;15:24406. doi: 10.1038/s41598-025-00012-7 (PMC12238603; doi:10.1038/s41598-025-00012-7)
Supplement: Supplementary file 1 — Supplementary Material 1 [file 41598_2025_12_MOESM1_ESM.docx]

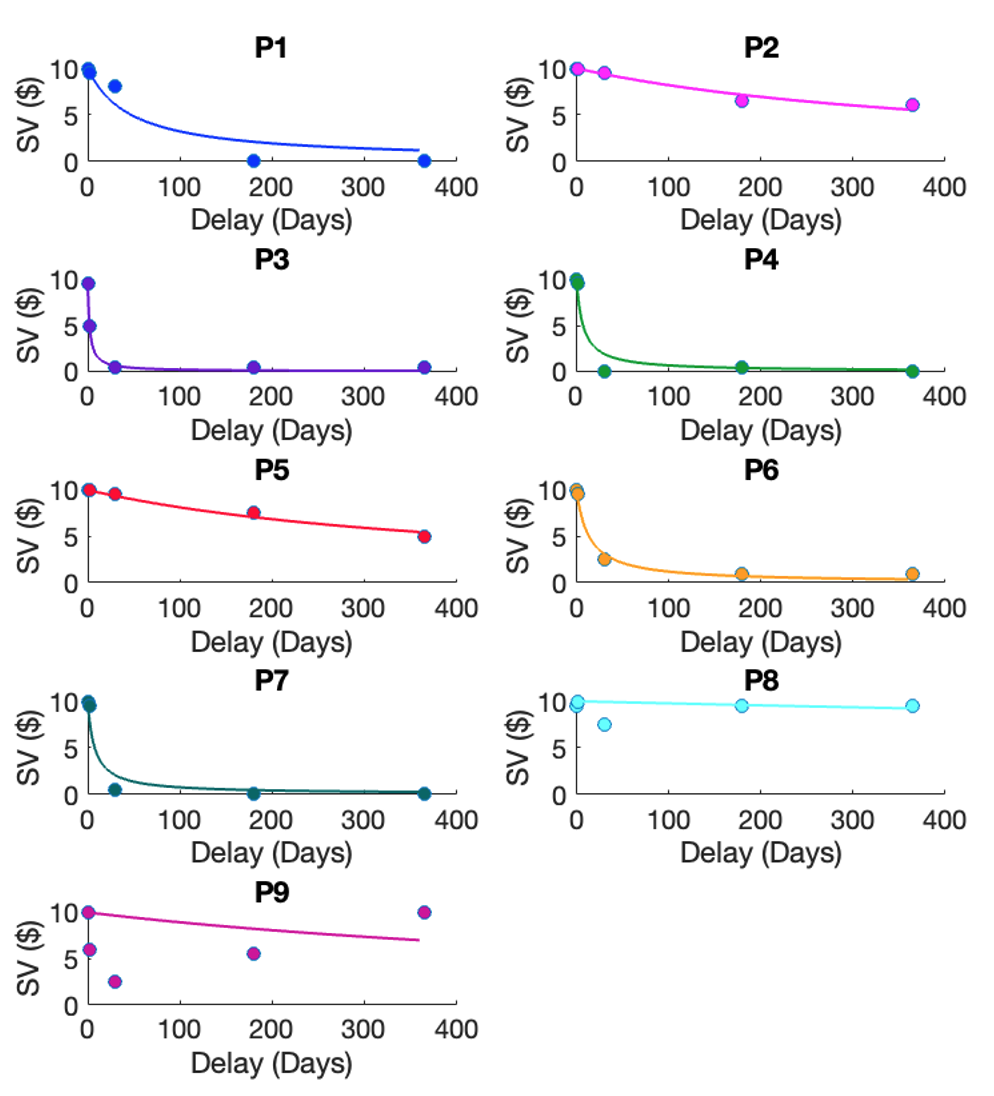


**Supplementary Figure 1**. Discounting curves for each participant (solid line) fit to indifference points (point at which at which y axis $ value was similarly valued to $10 at x axis delay; dots). Discounting curves were fit to indifference points using a one-parameter hyperbolic model (see Calculation of Participant-Specific Discounting Rate, Methods). Discounting curves with steeper slopes indicate that an individual rapidly reduces the subjective value (y axis) of a large, potential reward with increasing delay (x axis). Colors correspond to those used in Figure 1.


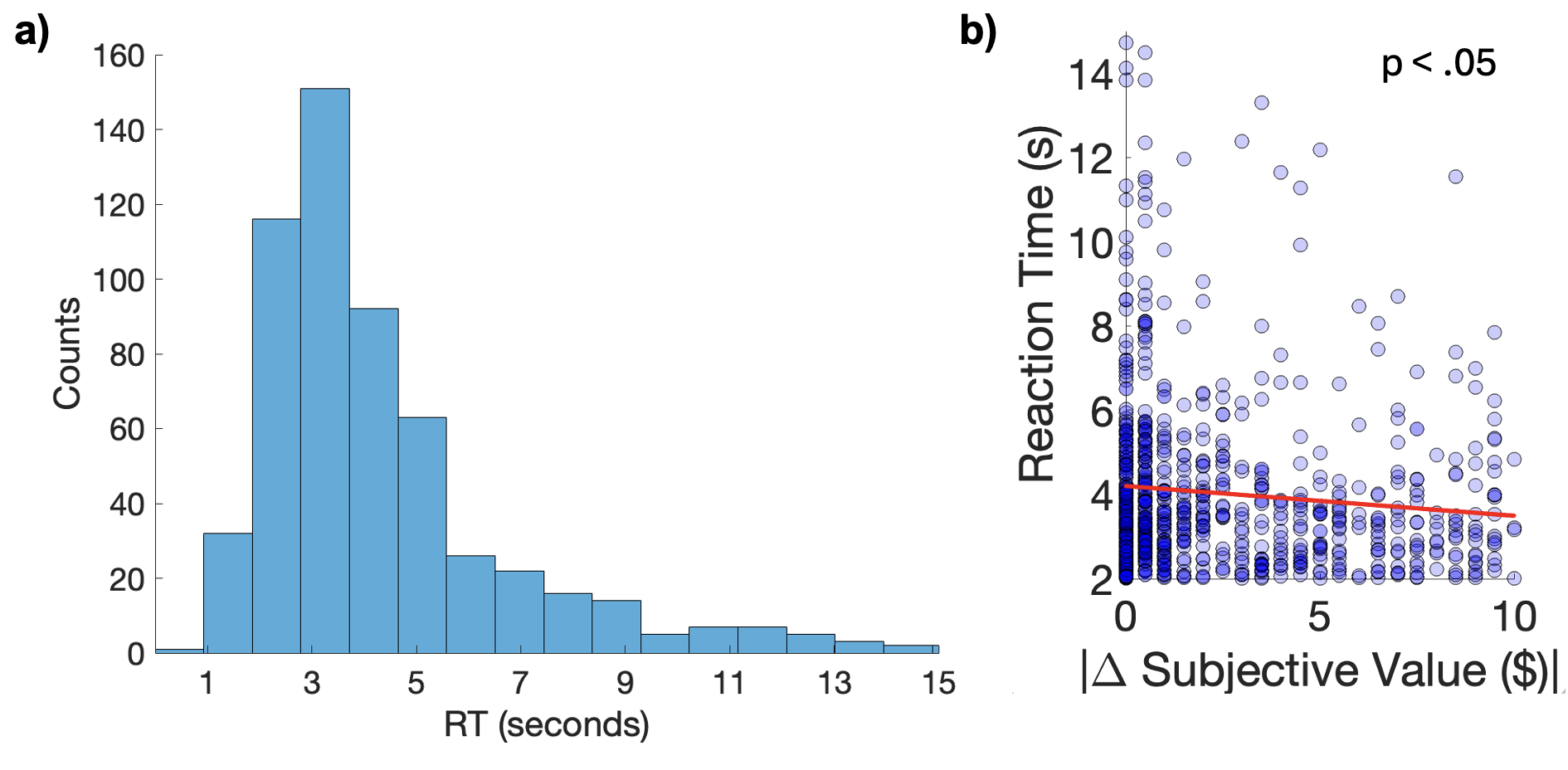


**Supplementary Figure 2.** Distribution of Observed Reaction Times and Subjective Values. a) Histogram of observed reaction times (seconds (s) across all trials across all participants. b) reaction time in seconds (s) across trials and the corresponding absolute value of difference in subjective value between offered options across all trials and subjects. Superimposed best fit linear function modeling the relationship between subjective value difference and RT. p = .036; Linear mixed effects model with fixed effect of subjective value difference on reaction time and random effect of subject.


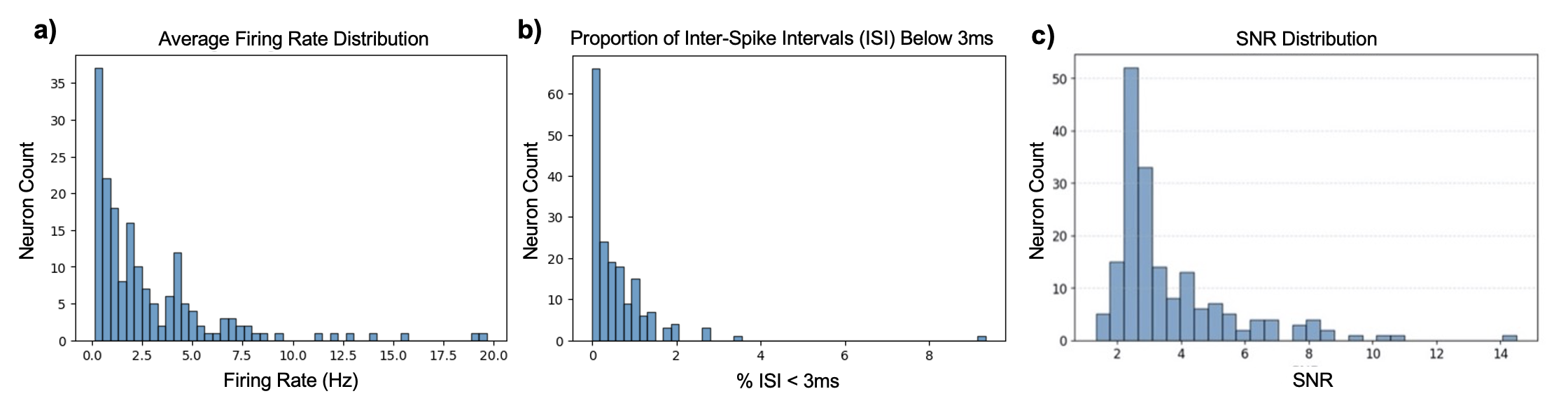


**Supplementary Figure 3**. Spike-sorting quality metrics. (**a**) Average firing rate. (**b**) Proportion of inter-spike intervals (ISI) below 3 ms. (**c**) Signal to noise ratio (SNR) defined as the maximum unit amplitude of the average waveform for each sorted unit divided by three average standard deviations of the background noise.


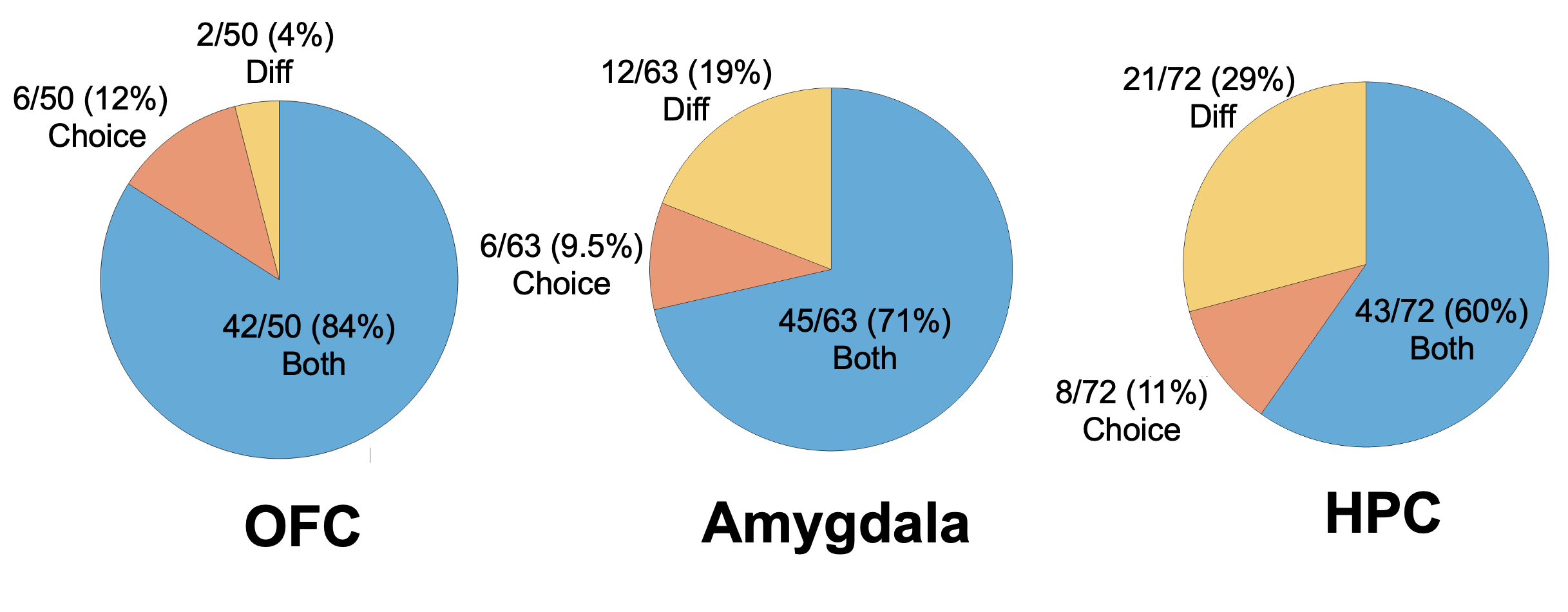


**Supplementary Figure 4**. **Unit Selectivity**. Number of units that significantly predicted choice, difficulty, or both over the number of units that predicted choice or difficulty within the orbitofrontal cortex (OFC), amygdala or hippocampus (HPC).


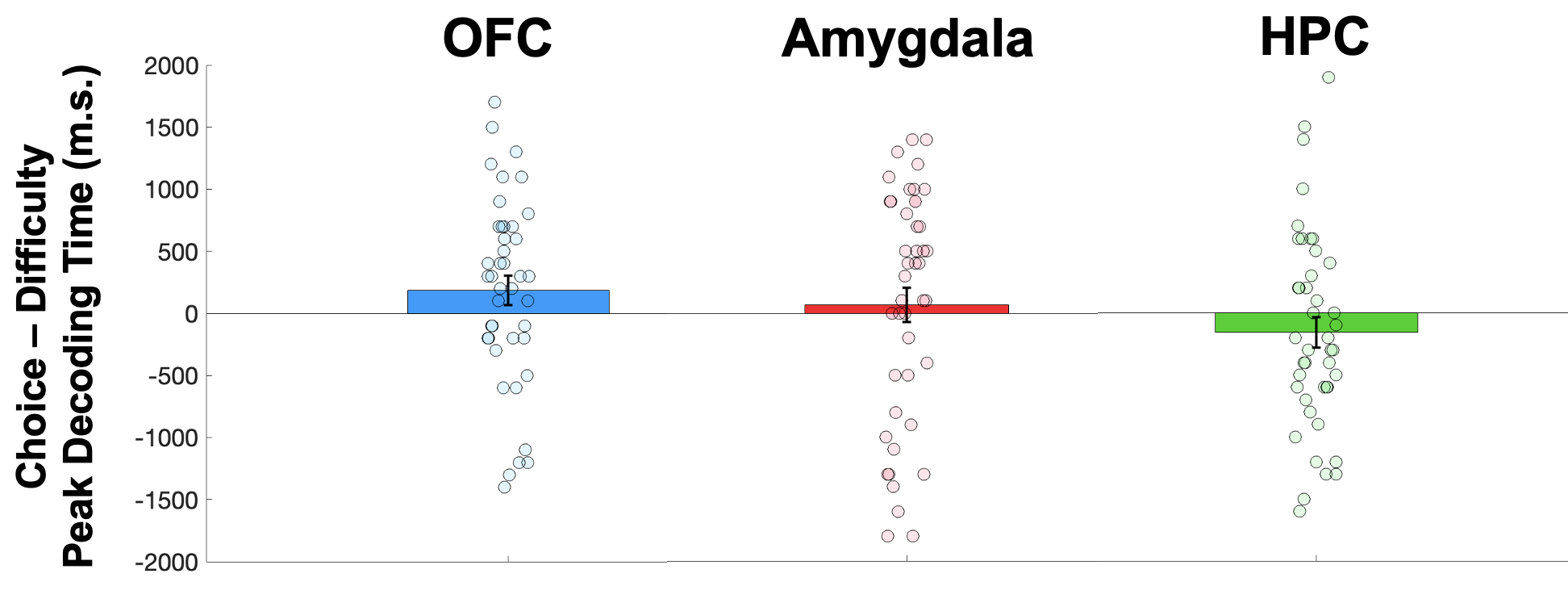


**Supplementary Figure 5**. **Peak Decoding Time Differences in Units that Encoded Choice and Difficulty**. Average millisecond (m.s.) difference in peak (time at which decoding accuracy prior to decision onset was highest prior to choice; Methods) decoding of choice and difficulty for individual units across n = 9 participants that decoded both choice and difficulty (Supplementary Figure 1) in the orbitofrontal cortex (OFC; blue; n = 42 units, choice mean = -952.38 ms +/- 14.90 s.e.m; difficulty mean = -1138.10 ms +/- 12.92 s.e.m; p = .073), amygdala (red; n = 45 units, choice mean = -1000.00 ms +/- 13.25 s.e.m; difficulty mean = -1068.90 ms +/- 13.12 s.e.m; p = .604) and the hippocampus (HPC; green; n = 43 units, choice mean = -1116.30 ms +/- 13.80 s.e.m; difficulty mean = -960.47 ms +/- 14.68 s.e.m; p = .159). Positive values indicate peak decoding accuracy of difficulty occurring before peak decoding time of choice and vice versa for negative values. Wilcoxon signed rank test of choice vs. difficulty peak decoding times did not achieve significance in any region (Methods).


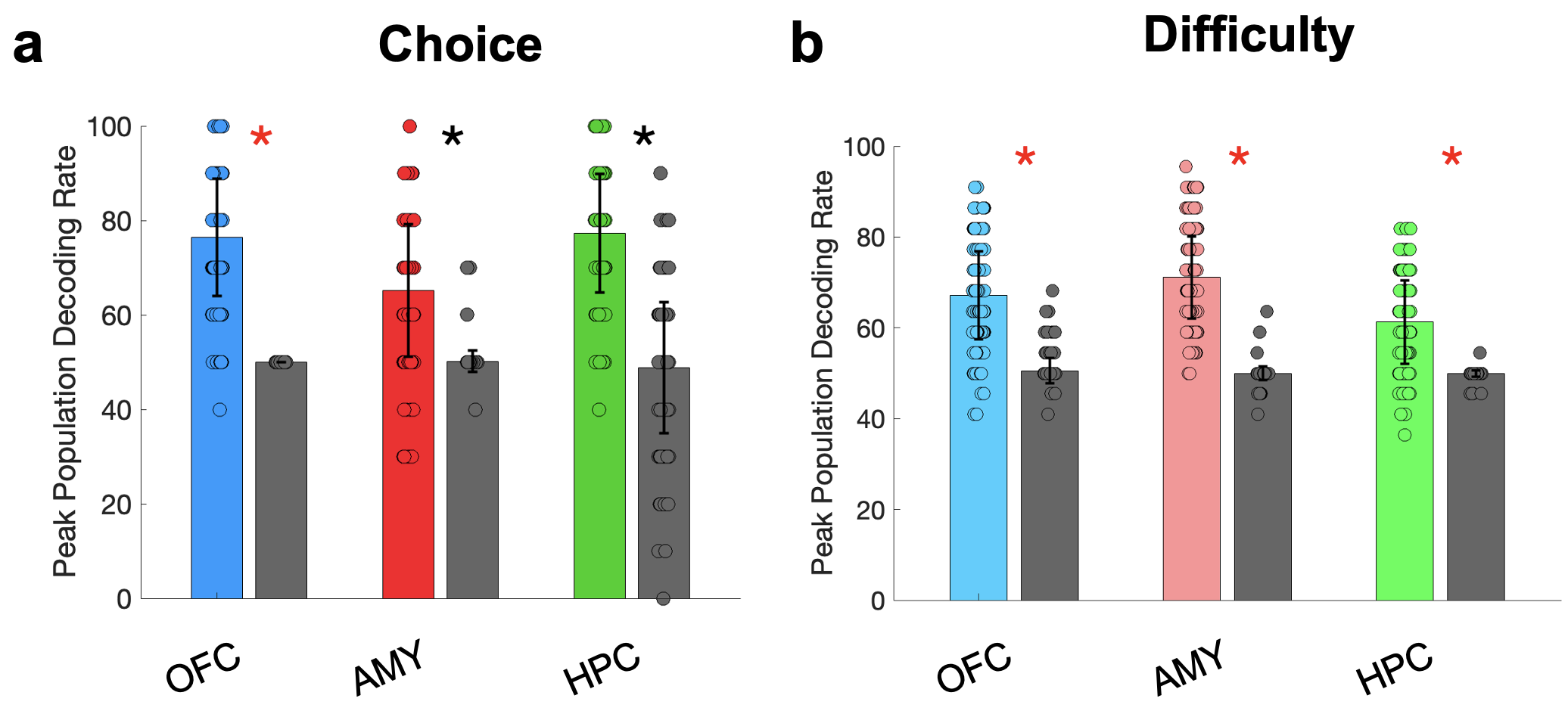


**Supplementary Figure 6. Multiregional Choice and Difficulty Peak Population Decoding.** **a)** Average accuracy +/- standard deviation (s.d) in the orbitofrontal cortex (OFC; blue), amygdala [red] and hippocampus [HPC; green] of a peak population decoder trained to predict trial choice prior to decision onset (Methods). Gray bar represents the corresponding average accuracy of a chance decoder trained on identical data, but with shuffled labels (Methods). Asterisks indicate above chance decoding. black * = p <.05, red * = p <.01. **b)** same as **a** but for a decoder trained to predict decision difficulty prior to choice (Methods).


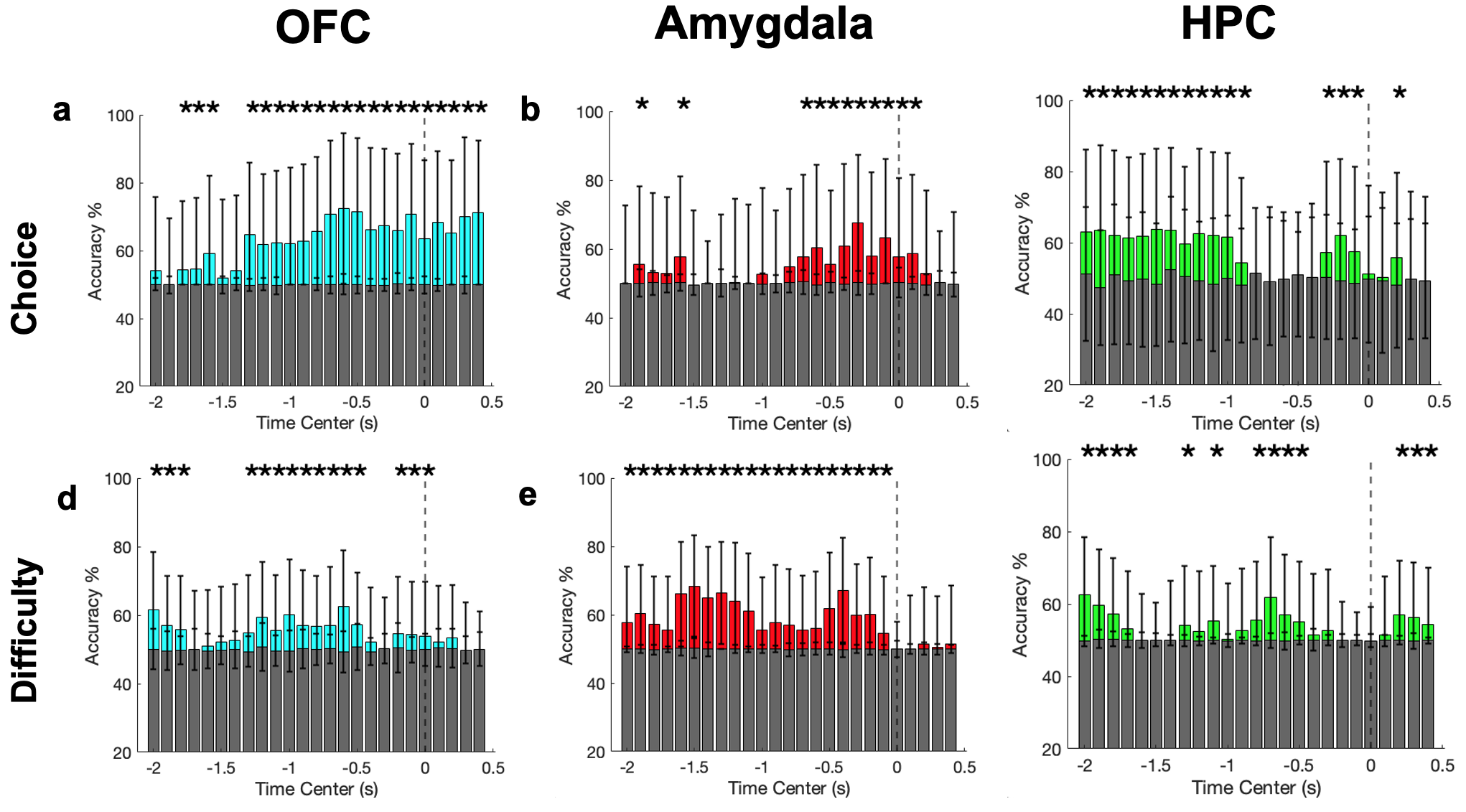


**Supplementary Figure 7. Multiregional Choice and Difficulty Peak Population Decoding**. **a)** Average accuracy +/- standard deviation [s.d] in the orbitofrontal cortex [OFC; blue], amygdala [red] and hippocampus [HPC; green] of a population decoder trained to predict trial choice (top row; **a-c)** or difficulty (bottom row; **d-f)** prior to decision onset (Methods). Gray bar represents the corresponding average accuracy of a chance decoder trained on identical data, but with shuffled labels (Methods). Asterisks indicate above chance decoding. * = p <.05 using permutation testing.


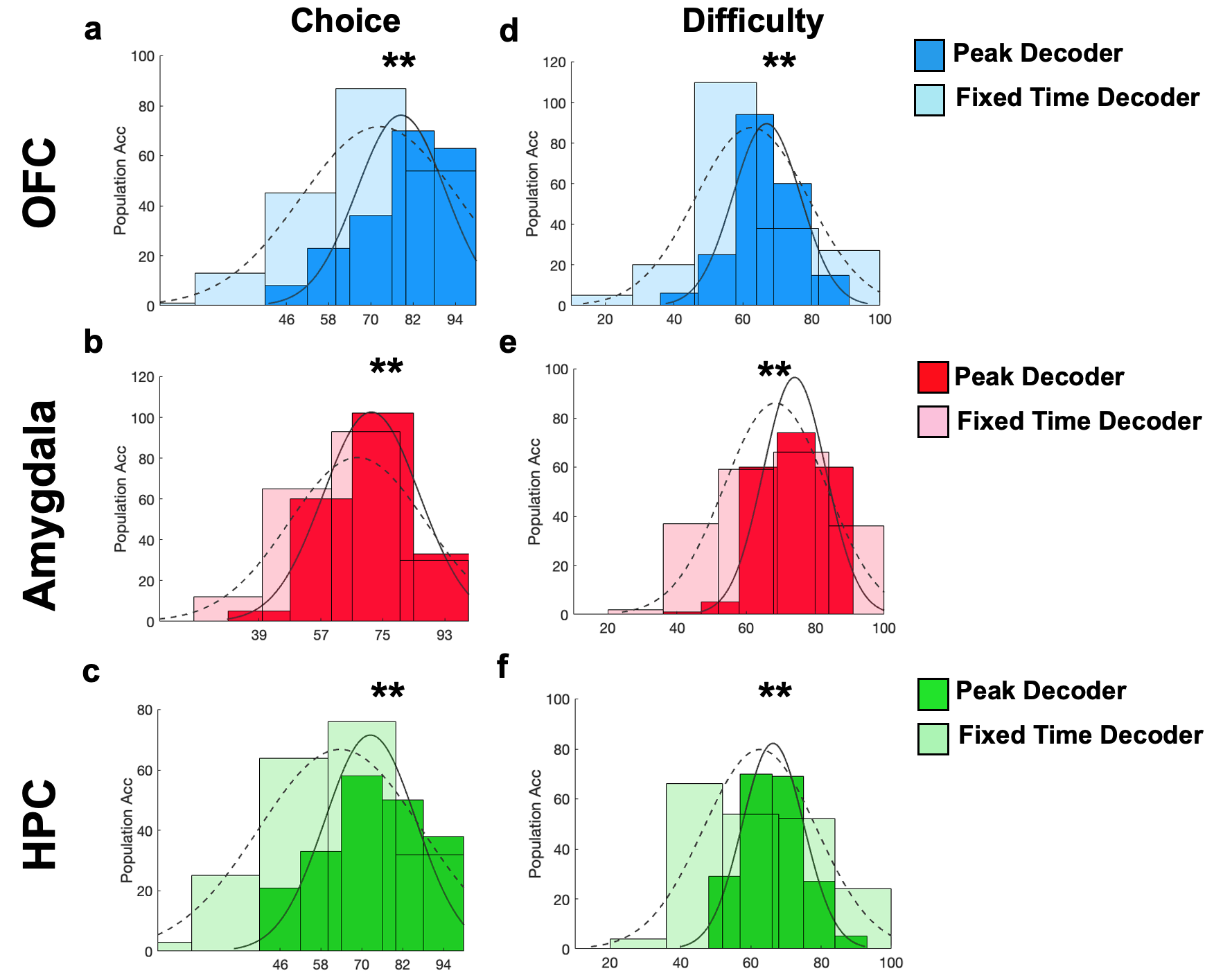


**Supplementary Figure 8. Improved Population Decoding with Peak Accuracy Decoding Approach a-c)** Histogram of decoding performance across 200 cross validations from a model built using average activity from units (features) in 500 ms centered where decoding accuracy peaked (darker shade of all colors; Peak Decoder; Methods) vs. a model using activity centered at the same time point across units when collective accuracy was highest (lighter shade of all colors; Fixed Time Decoder; Methods) during decoding of choice (left) and difficulty (right). Units were isolated from **a, d)** the orbitofrontal cortex [OFC], **b, e)** the amygdala and **c, f)** the hippocampus (HPC). * = p<.05, ** = p<.01 using permutation testing (Methods). **a)** OFC choice mean +/- standard deviation (s.d.) = Peak: 78.6 +/- 12.56; Fixed: 72.50 +/- 22.27. **d)** OFC difficulty mean +/- s.d = Peak: 66.97 +/- 9.79; Fixed: 62.63 +/- 16.38. **b)** Amygdala choice mean +/- s.d = Peak: 71.7 +/- 13.99; Fixed: 67.63 +/- 19.86. **e)** Amygdala difficulty mean +/- s.d = Peak: 74.06 +/- 9; Fixed: 68.50 +/-14.83. **c)** HPC choice mean +/- s.d = Peak: 72.55+/- 13.38; **f)** Fixed: 63.250 +/- 24.02. HPC difficulty mean +/- s.d = Peak: 66.32 +/- 8.73;Fixed: 62.65 +/- 15.99.


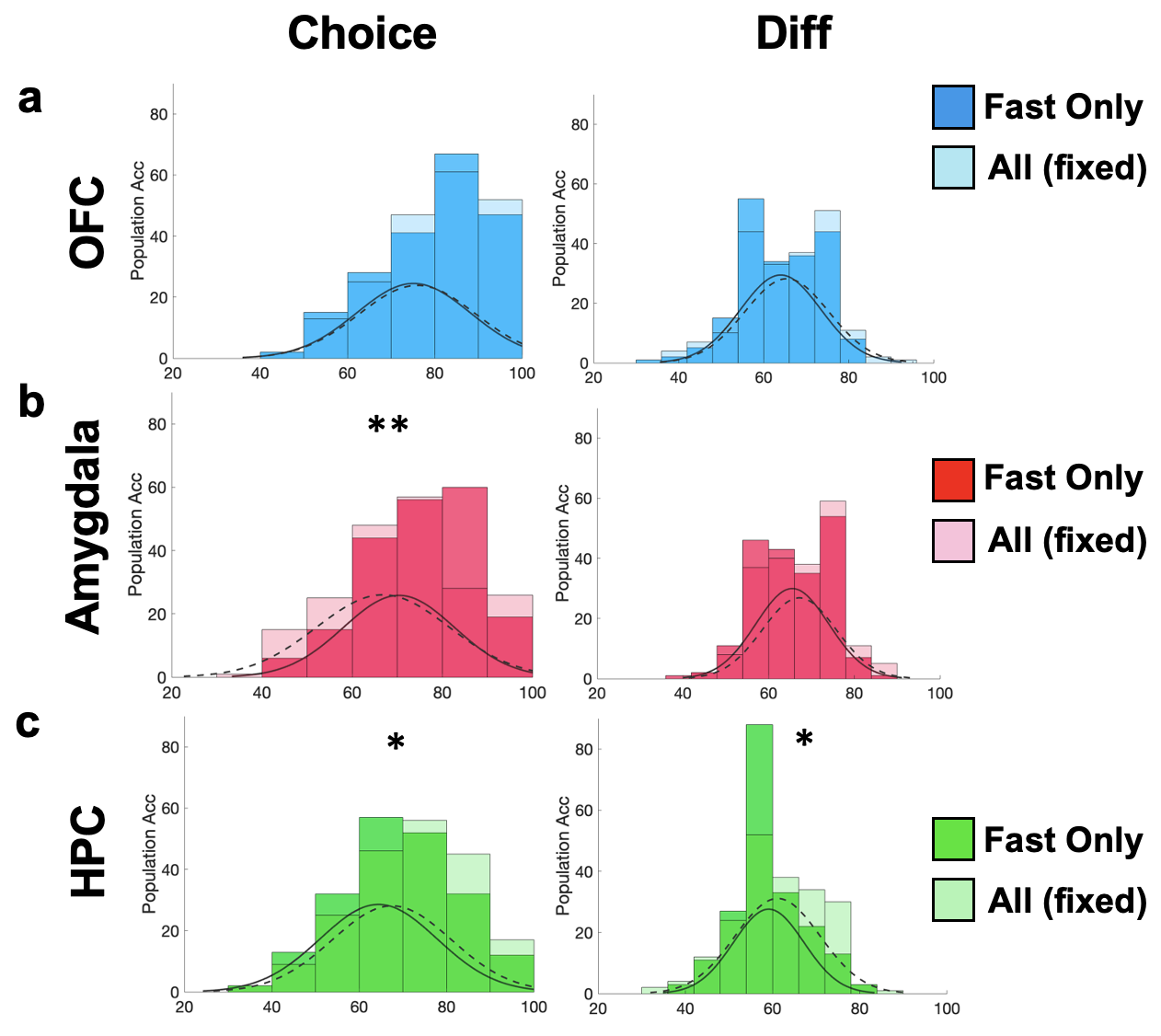


**Supplementary Figure 9. Altered Decoding in Fast Discounter Model.** **a-c)** Histogram of decoding performance across 200 cross validations from a model built using average activity from units (features) from participants with fast discounting rates (darker shade of all colors; fast only) vs. a model built using a matched number of randomly selected units from all participants (lighter shade of all colors; All (fixed)) during decoding of choice (left) and difficulty (diff; right). Units were located in: **a)** the orbitofrontal cortex (OFC, fast only choice mean +/- standard deviation (s.d.) = 75.15% +/- 13.03, all (fixed) choice mean +/- s.d. = 76.05% +/- 13.37, p = .451; fast only diff mean +/- s.d. = 64.03 % +/- 9.48, all (fixed) diff mean +/- s.d. = 65.45% +/- 9.90, p = .130), **b)** the amygdala (fast only choice mean +/- s.d. = 70.40% +/- 12.35, all (fixed) choice mean +/- s.d. = 66.85% +/- 14.72, p = .009; fast only diff mean +/- s.d. = 65.55% +/- 8.52, all (fixed) diff mean +/- s.d. = 67.18% +/- 8.61, p = .054) and **c)** the hippocampus (HPC, fast only choice mean +/- s.d. = 59.13% +/- 8.08; all (fixed) choice mean +/- s.d. = 61.18 % +/- 9.74; p = .024). * = p<.05, ** = p<.01 using permutation testing (Methods).


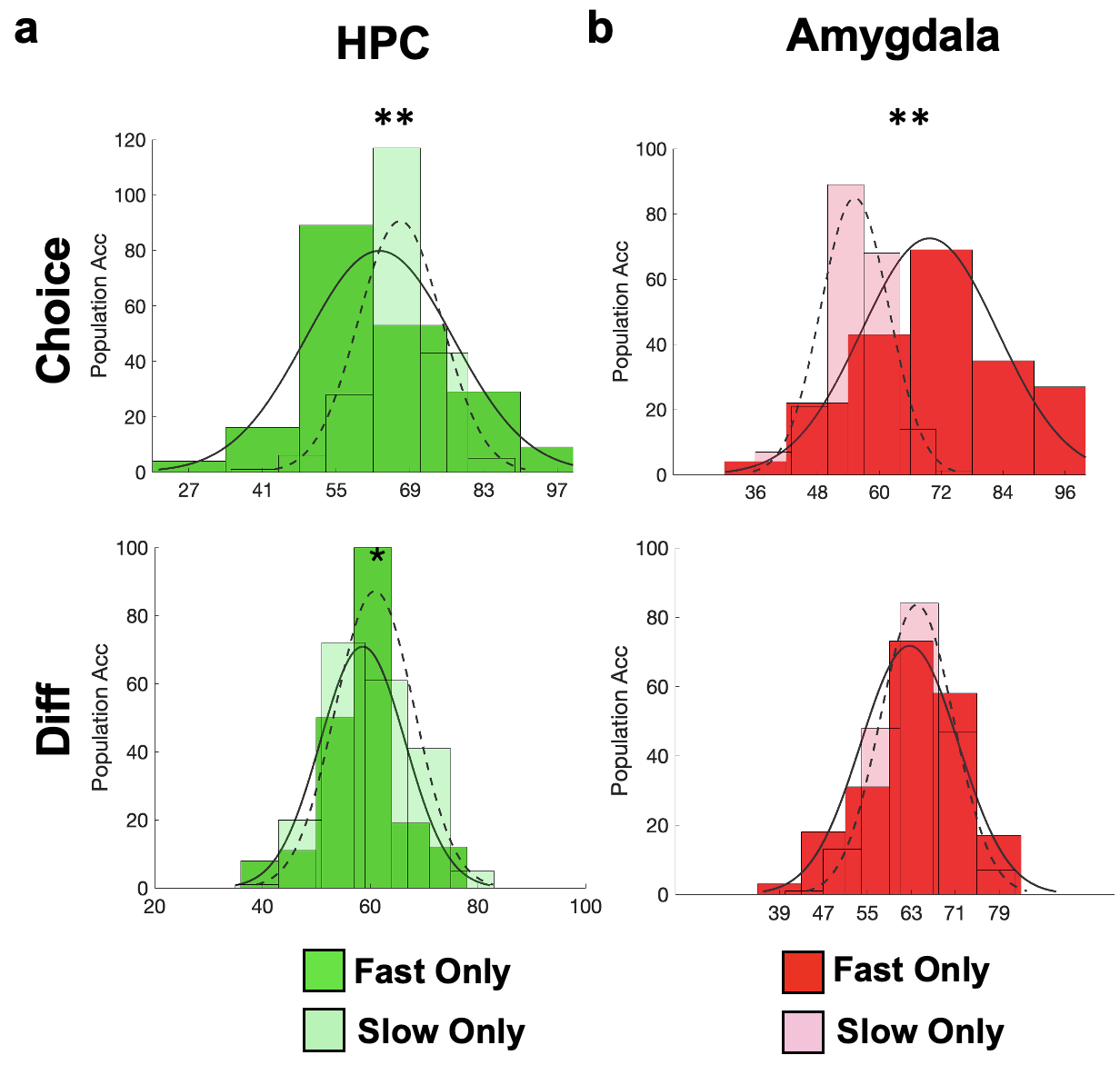


**Supplementary Figure 10. Differential Amygdala vs. Hippocampal Variable Encoding in Slow and Fast Discounter Models**. **a-b)** Histogram of decoding performance across 200 cross validations from a model built using average activity from units (features) from participants with fast discounting rates (darker shade of red/green; Fast Only) vs. a model built using a matched number of units from all participants with slow discounting rates (lighter shade of red/green; Slow Only) during decoding of choice (top row) and difficulty (right). Units were isolated from **a)** the hippocampus (HPC, fast only choice mean +/- standard deviation (s.d.) = 63.20% +/- 13.99, slow only choice mean +/- s.d. = 67.13% +/- 7.92, p < .001; fast only diff mean +/- s.d. = 58.6% +/- 7.88, slow only diff mean +/- s.d. = 60.90 % +/- 7.32, p = .030) and **b)** the amygdala (fast only choice mean +/- s.d. = 69.75 % +/- 13.20, slow only choice mean +/- s.d. = 55.25% +/- 6.57, p < .001; fast only diff mean +/- s.d. = 62.70% +/- 8.89, slow only diff mean +/- s.d. = 64.03% +/- 6.68, p =.247). * = p<.05, ** = p<.01 using permutation testing (Methods)


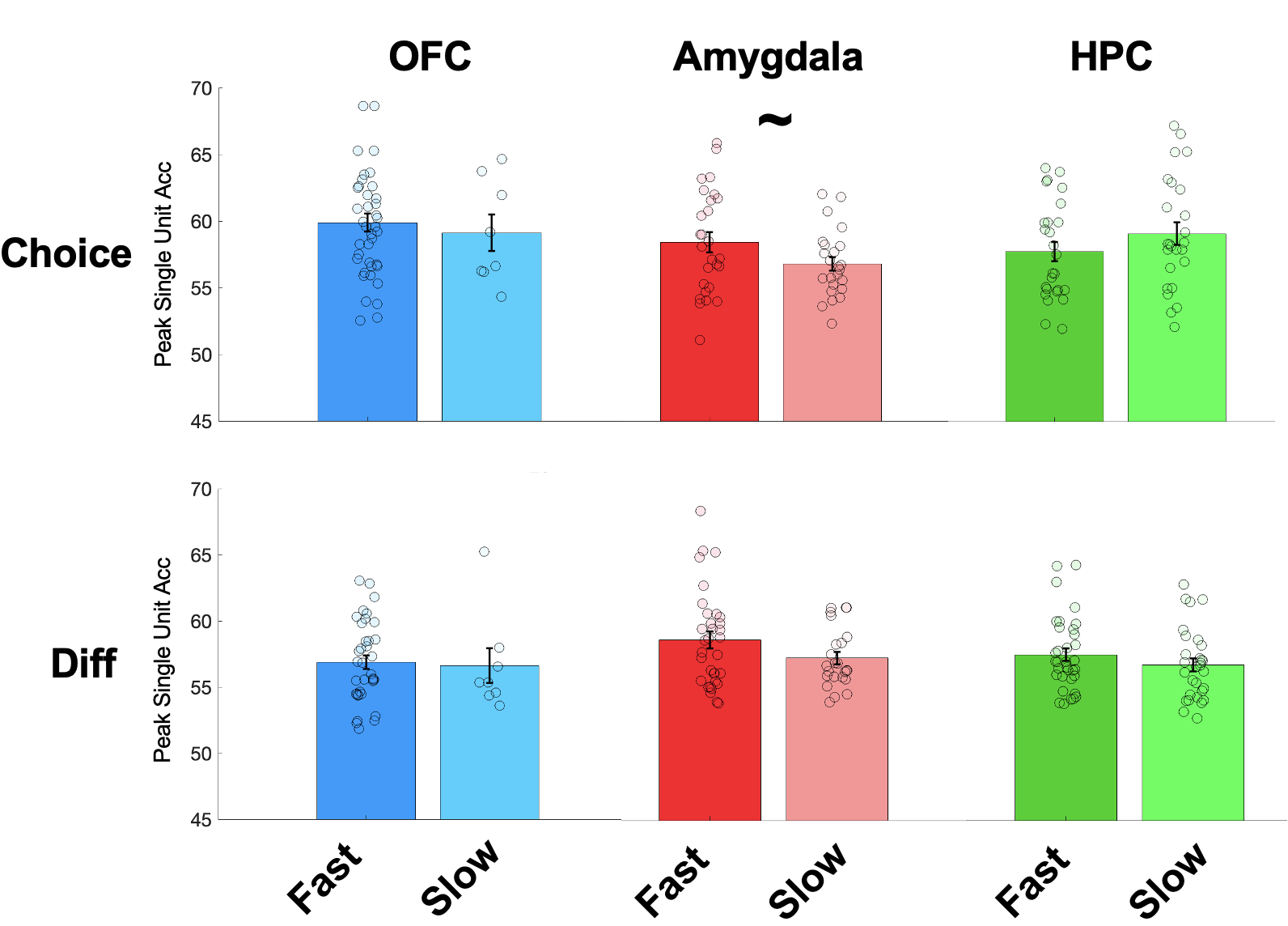


**Supplementary Figure 11. Single Unit Peak Decoding Accuracy In Slow and Fast Discounters**. Average peak single unit choice (top row) and difficulty (Diff; bottom row) decoding accuracy in slow and fast discounters (Methods) in the orbitofrontal cortex (OFC; blue) amygdala (red) and hippocampus (HPC; green). ~ = p <.1 using a linear mixed effects model (Methods). OFC average fast discounter unit peak choice decoding accuracy +/- standard error of the mean (s.e.m.) = 59.88% +/- .68, average slow discounter unit peak choice decoding accuracy +/- s.e.m. = 59.12% +/- 1.37; p = .359. OFC average fast discounter unit peak diff decoding accuracy +/- s.e.m.= 56.88% +/- .51, average slow discounter unit peak diff decoding accuracy +/- s.e.m.= 56.62% +/- 1.32 ; p = .885. Amygdala average fast discounter unit peak choice decoding accuracy +/- s.e.m. = 58.56% +/- .75, average slow discounter unit peak choice decoding accuracy +/- s.e.m. = 56.92% +/- .51; p = .085. Amygdala average fast discounter unit peak diff decoding accuracy +/- s.e.m.= 58.23% +/- .61, average slow discounter unit peak diff decoding accuracy +/- s.e.m.= 56.90% +/- .44; p = .215. HPC average fast discounter unit peak choice decoding accuracy +/- s.e.m. = 57.90% +/- .73, average slow discounter unit peak choice decoding accuracy +/- s.e.m. = 59.26 +/- .86 ; p = .897. HPC average fast discounter unit peak diff decoding accuracy +/- s.e.m. = 57.12% +/- . 46, average slow discounter unit peak diff decoding accuracy +/- s.e.m. = 56.39%+/- .47; p = .367.


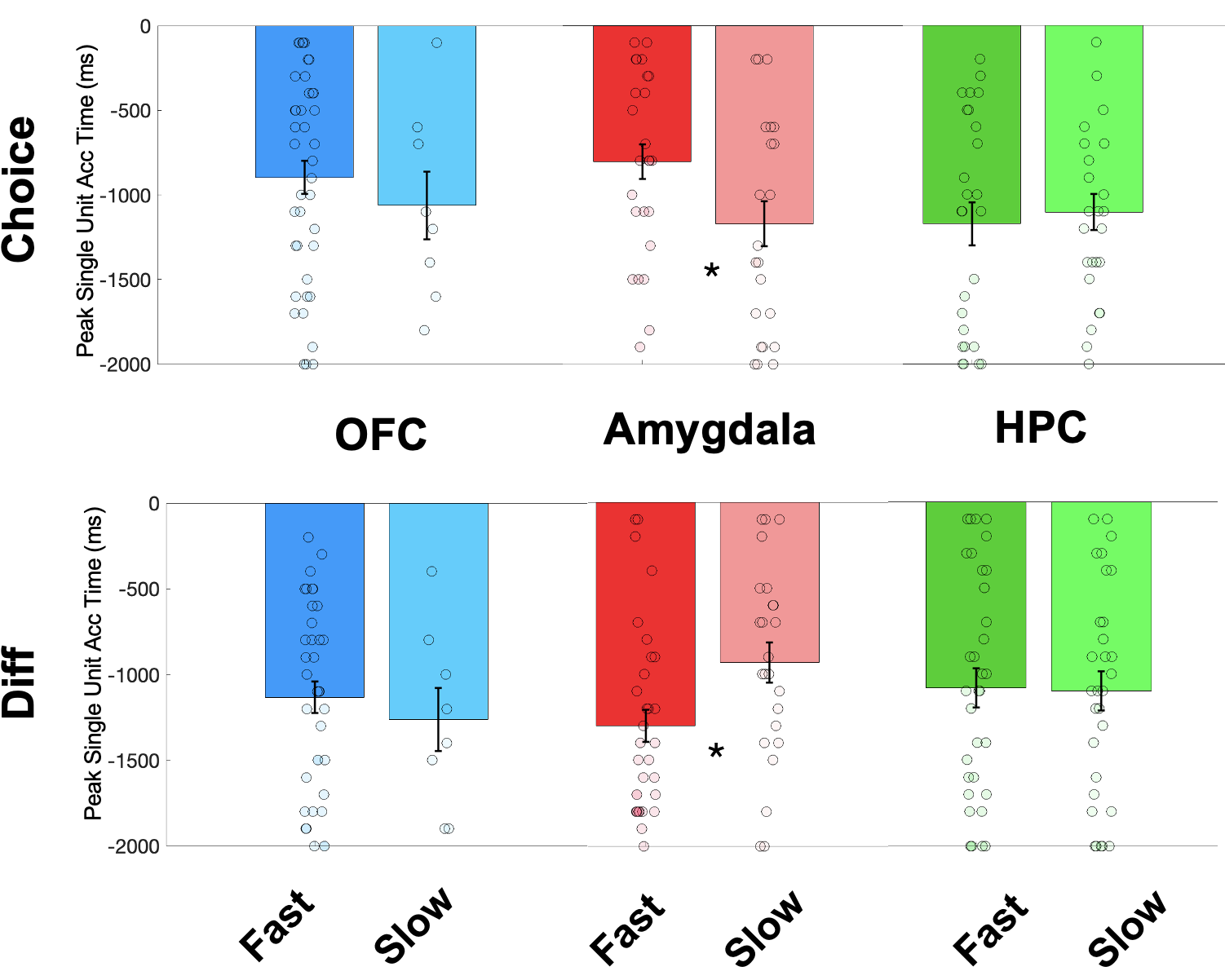


**Supplementary Figure 12. Peak Decoding Time Differences in Slow and Fast Discounters.** Average millisecond (m.s.) peak decoding time (time at which decoding accuracy was highest prior to choice; Methods) of choice (top row) and difficulty (Diff; bottom row) for individual units across slow and fast discounters (Methods) in the orbitofrontal cortex (OFC; blue), amygdala (red) and the hippocampus (HPC; green). * = p <.05 using a linear mixed effects model (Methods). OFC average fast discounter unit peak choice decoding time +/- standard error of the mean (s.e.m.) = -897.5 m.s. +/- 199.94, average slow discounter unit peak choice decoding time +/- s.e.m.= -1062.5 m.s +/- 97.96; p =.501. OFC average fast discounter unit peak diff decoding time +/- s.e.m. = -1133.3 m.s. +/- 92.50, average slow discounter unit peak diff decoding time +/- s.e.m. = -1262.5 m.s. +/- 185.10 ; p =. 551. Amygdala average fast discounter unit peak choice decoding time +/- s.e.m. = -803.70 m.s. +/- 102.04, average slow discounter unit peak choice decoding time +/- s.e.m. = -1170.8 m.s. +/- 132.89 ; p = .031. Amygdala average fast discounter unit peak diff decoding time +/- s.e.m. = -1300.00 m.s. +/- 94.15, average slow discounter unit peak diff decoding time +/- s.e.m. = -933.33 m.s. +/- 115.88; p = .016. HPC average fast discounter unit peak choice decoding time +/- s.e.m.= -1173.10 m.s +/- 126.44, average slow discounter unit peak choice decoding time +/- s.e.m. = -1104.00 m.s +/- 17.31; p = .679. HPC average fast discounter unit peak diff decoding time +/- s.e.m. = -1082.40 m.s. +/- 113.17, average slow discounter unit peak diff decoding time +/- s.e.m. = -1100 m.s. +/- 114.47; p = .959.


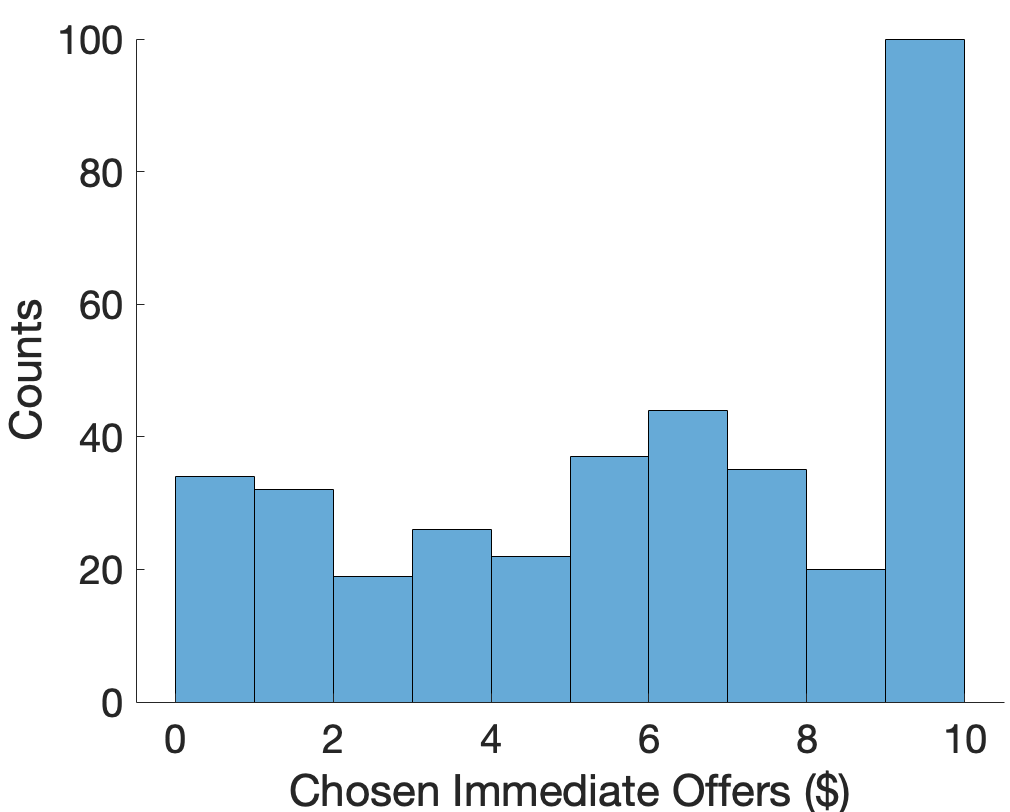


**Supplementary Figure 13.** Number of trials that an immediate offer of a specified value less than $10 was chosen over $10 available at a specified delay.


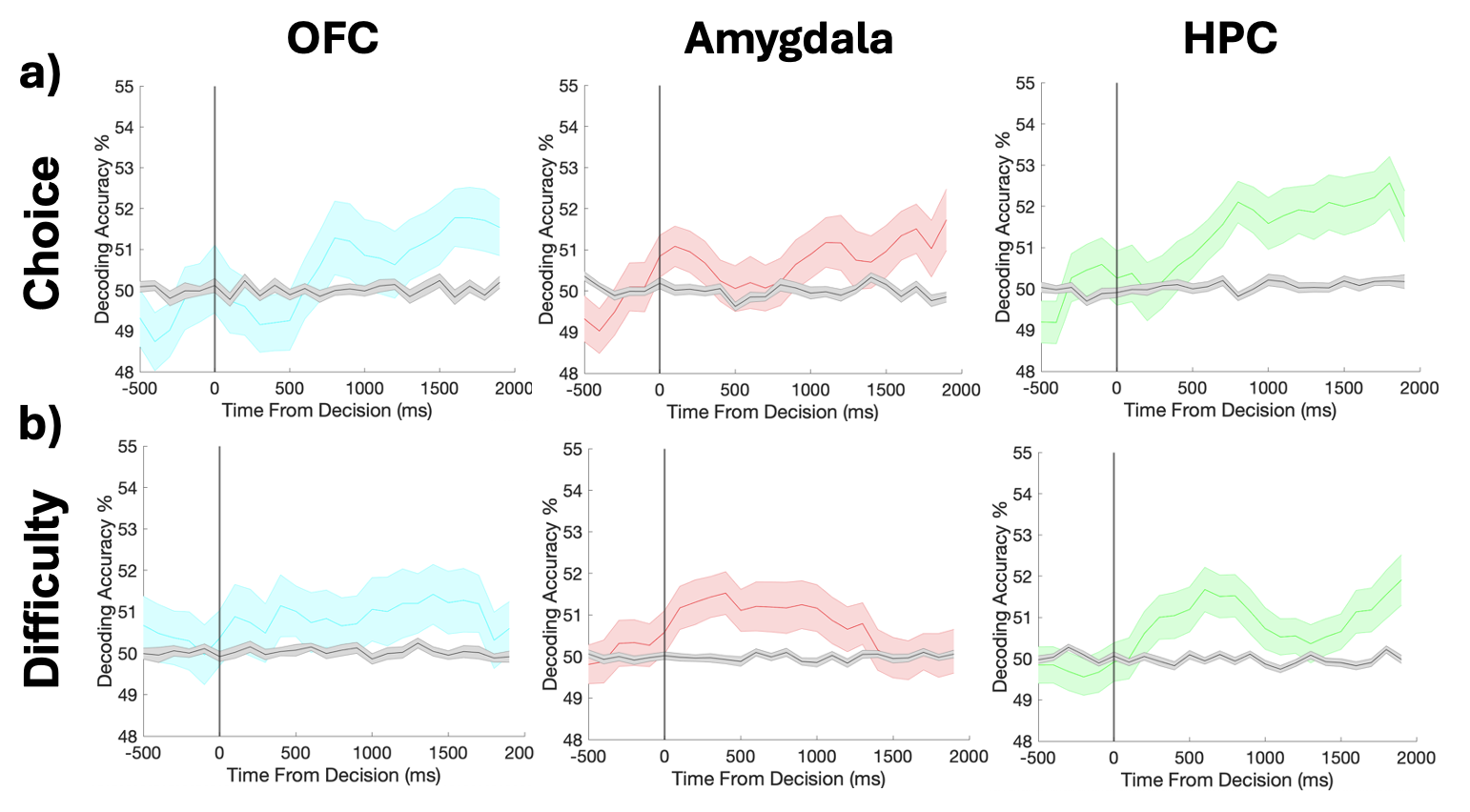


**Supplementary Figure 14.** Cue Aligned Decoding of Intertemporal in Human Single Neurons in the Orbitofrontal Cortex, Amygdala, and Hippocampus a) The average decoding accuracy +/- s.e.m of choice selective units from the OFC (left), amygdala (AMY; middle) and hippocampus (HPC; right) aligned to prompt presentation. Vertical line represents onset of trial (Figure 1b) with activity preceding activity incorporating data during fixation cross presentation **b)** Same as **a** but for difficulty encoding units that predicted whether a choice being made was hard or easy.


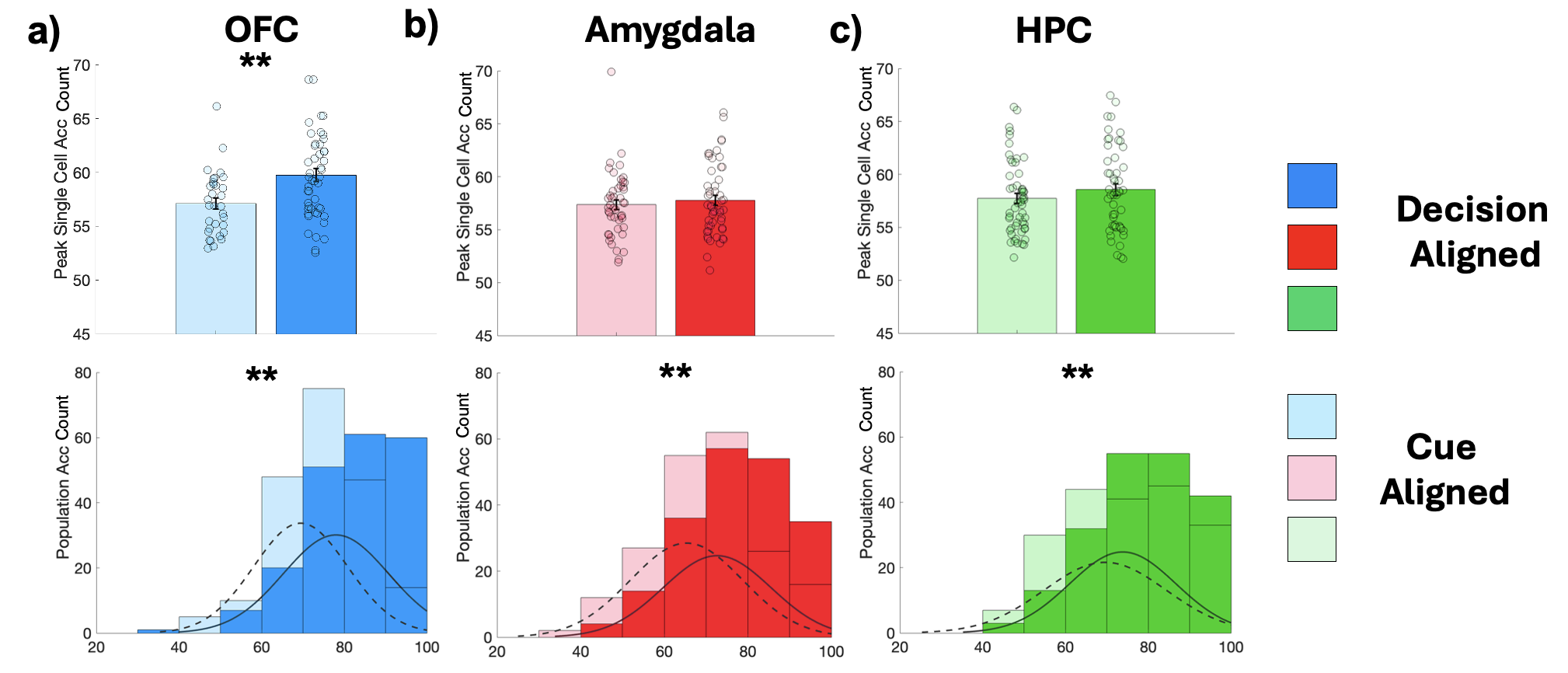


**Supplementary Figure 15.** Enhanced decoding of choice during the peri-decision period. Average peak single unit activity during decoding of choice using activity two seconds after cue onset (lighter shade of all colors) and before decision (darker shade of all colors **a)** OFC (blue; 32 peri-cue units, 48 peri-decision units), [Top; average unit peak choice decoding accuracy during cue onset (“Cue Aligned”; light blue) +/- standard error of the mean (s.e.m.) = 57.12% +/- 0.52 , average unit peak choice decoding accuracy during decision onset (“Decision Aligned”; Dark blue) +/- s.e.m.= 59.75% +/- 0.61 p = 0.004], **b)** amygdala (red; Choice: 46 peri-cue units, 51 peri-decision units) [Top; average unit peak choice decoding accuracy during cue onset (“Cue Aligned”; light red) +/- standard error of the mean (s.e.m.) = 57.36% +/- 0.46, average unit peak choice decoding accuracy during decision onset (“Decision Aligned”; Dark red) +/- s.e.m. = 57.78% +/- 0.47 p = 0.362] and **c)** HPC (green; Choice: 54 peri-cue units, 51 peri-decision units) [Top; average unit peak choice decoding accuracy during cue onset (“Cue Aligned”; light green) +/- standard error of the mean (s.e.m.) = 57.73% +/- 0.47, average unit peak choice decoding accuracy during decision onset (“Decision Aligned”; Dark green) +/- s.e.m. = 58.56% +/- 0.56 p = 0.266] . * = p < .05, ** = p<.01 using linear mixed effect models. Histogram of decoding performance across 200 cross validations from choice (darker shade of all colors) and difficulty (lighter shade of all colors) decoding models built using activity from units (features) that significantly encoded choice and difficulty, respectively, in the **a)** orbitofrontal cortex (OFC; blue) [Bottom; average population decoding accuracy during cue onset (“Cue Aligned”; light blue) +/- standard error of the mean (s.e.m.) = 69.45 +/- 11.35, average population decoding accuracy during decision onset (“Decision Aligned”; Dark blue) +/- s.e.m.= 78.00% +/- 12.72 p = 0.001], **b)** amygdala (red) [Bottom; average population decoding accuracy during cue onset (“Cue Aligned”; light red) +/- standard error of the mean (s.e.m.) = 65.35% +/- 13.44, average population decoding accuracy during decision onset (“Decision Aligned”; Dark red) +/- s.e.m.= 72.65% +/- 12.94 p =0.001 ] and **c)** hippocampus (HPC; green) [Bottom; average population decoding accuracy during cue onset (“Cue Aligned”; light green) +/- standard error of the mean (s.e.m.) = 69.60% +/- 14.76, average population decoding accuracy during decision onset (“Decision Aligned”; Dark green) +/- s.e.m.= 73.85% +/- 12.86 p = 0.001 ]. * = p < .05, ** = p<.01 using permutation testing


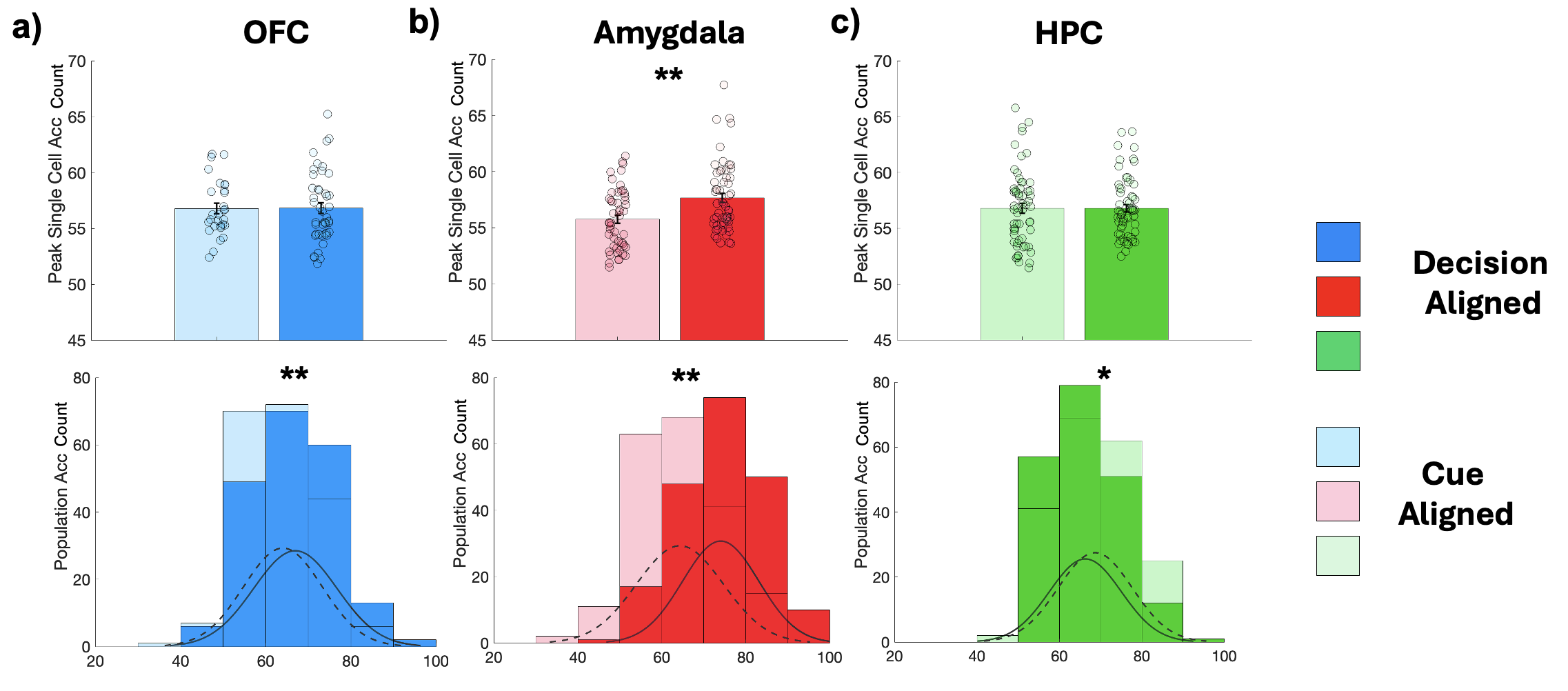


**Supplementary Figure 16.** Decoding of Decision Difficulty During the Cue vs. Decision Period. Average peak single unit activity during decoding of decision difficulty using activity two seconds after cue onset (lighter shade of all colors) and before decision (darker shade of all colors **a)** OFC (blue; 29 peri-cue units, 44 peri-decision units), [Top; average unit peak difficulty decoding accuracy during cue onset (“Cue Aligned”; light blue) +/- standard error of the mean (s.e.m.) = 56.79% +/- 0.46 , average unit peak difficulty decoding accuracy during decision onset (“Decision Aligned”; Dark blue) 56.83% +/- s.e.m.= 0.47 p = 0.936], **b)** amygdala (red; 50 peri-cue units, 57 peri-decision units) [Top; average unit peak difficulty decoding accuracy during cue onset (“Cue Aligned”; light red) +/- standard error of the mean (s.e.m.) = 55.76% +/- 0.379, average unit peak difficulty decoding accuracy during decision onset (“Decision Aligned”; Dark red) +/- s.e.m. = 57.67% +/- 0.41 p = <0.001], and **c)** HPC (green; 59 peri-cue units, 64 peri-decision units). [Top; average unit peak difficulty decoding accuracy during cue onset (“Cue Aligned”; light green) +/- standard error of the mean (s.e.m.) = 56.80% +/- 0.44, average unit peak difficulty decoding accuracy during decision onset (“Decision Aligned”; Dark green) +/- s.e.m. = 56.77% +/- 0.33 p = 0.974]. * = p < .05, ** = p<.01 using linear mixed effect models. Histogram of decoding performance across 200 cross validations from choice (darker shade of all colors) and difficulty (lighter shade of all colors) decoding models built using activity from units (features) that significantly encoded choice and difficulty, respectively, in the **a)** orbitofrontal cortex (OFC; blue) [Bottom; average population decoding accuracy during cue onset (“Cue Aligned”; light blue) +/- standard error of the mean (s.e.m.) = 64.04% +/- 9.26, average population decoding accuracy during decision onset (“Decision Aligned”; Dark blue) +/- s.e.m.= 66.97% +/- 9.79 p = 0.01], **b)** amygdala (red) [Bottom; average population decoding accuracy during cue onset (“Cue Aligned”; light red) 64.34% +/- 10.34 standard error of the mean (s.e.m.) =, average population decoding accuracy during decision onset (“Decision Aligned”; Dark red) 74.06% +/- 9.08 s.e.m.= p = 0.01] and **c)** hippocampus (HPC; green) [Bottom; average population decoding accuracy during cue onset (“Cue Aligned”; light green) +/- standard error of the mean (s.e.m.) = 68.70% +/- 8.98, average population decoding accuracy during decision onset (“Decision Aligned”; Dark green) 66.31% +/- 8.73 s.e.m.= p = 0.02]. * = p < .05, ** = p<.01 using permutation testing.


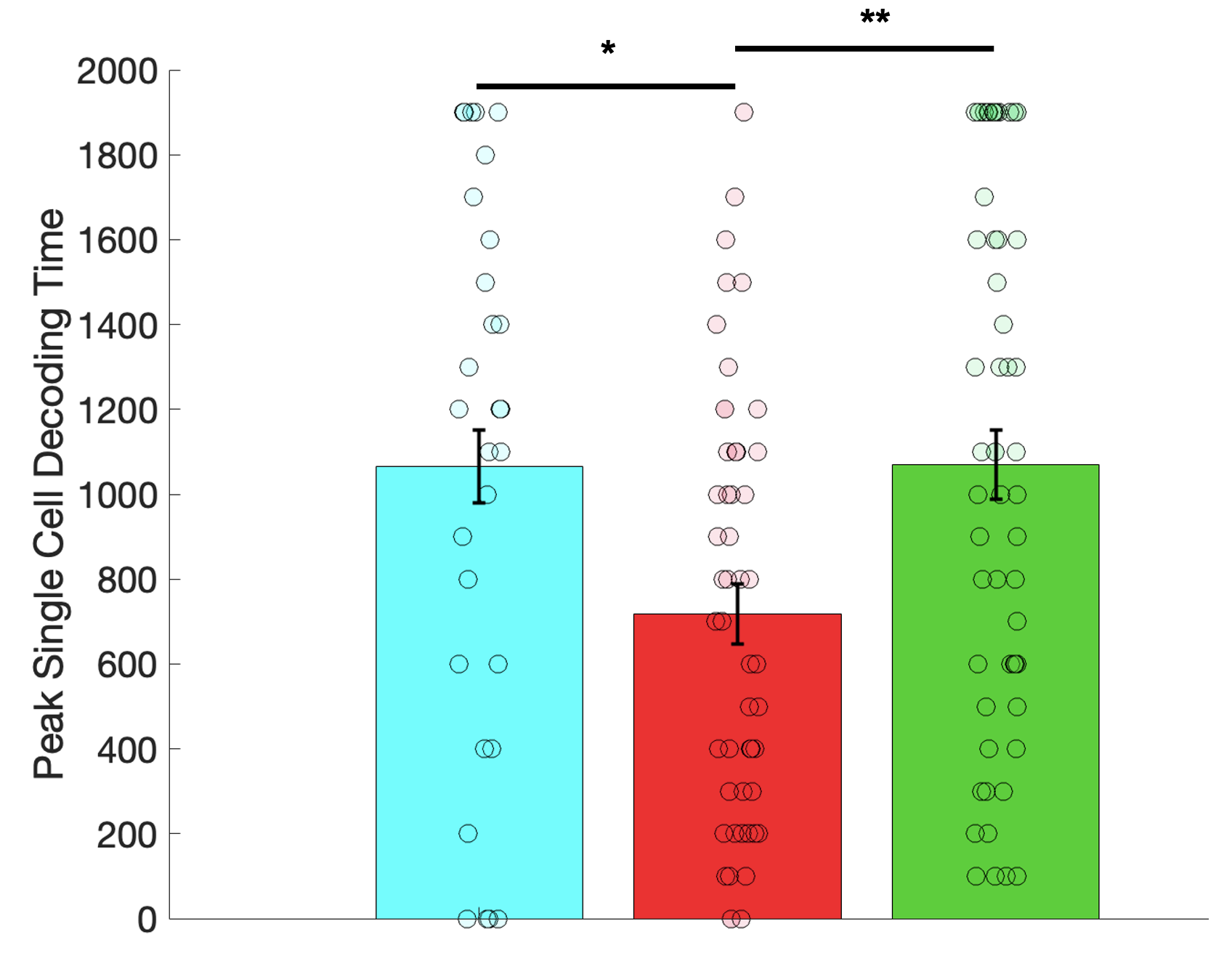


**Supplementary Figure 17.** Peak Decoding Time Differences in Cue-Aligned Encoding of Difficulty. Average millisecond (m.s.) peak (time at which decoding accuracy was highest within 2 seconds of cue presentation; Methods) decoding of difficulty for individual units across n = 9 participants that significantly decoded difficulty in the orbitofrontal cortex (OFC; blue; n = 32 units; mean peak decoding time +/- standard error of the mean = 1065 ms +/- 121.00), amygdala (red; n = 46 units; mean peak decoding time +/- standard error of the mean = 718 ms +/- 70.89) and the hippocampus (HPC; green; n = 59 units; mean peak decoding time +/- standard error of the mean = 1069 ms +/- 82.06). Wilcoxon rank sum test * = p <.05, ** = p<.01, Bonferroni corrected.


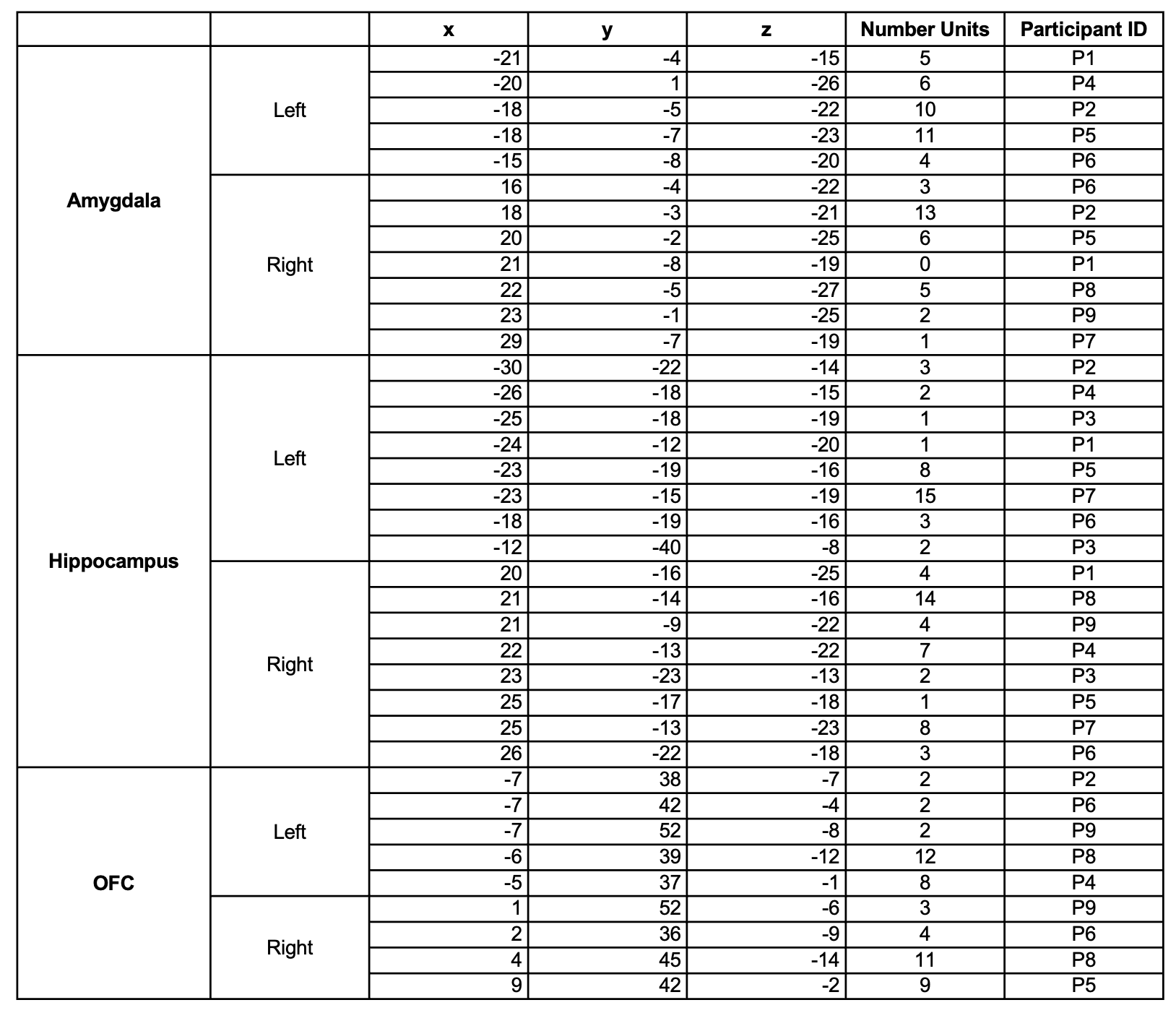


**Supplementary Table 1. Participant Electrode Locations.** The MNI coordinates and associated brain regions of microelectrode bundles for all included participants


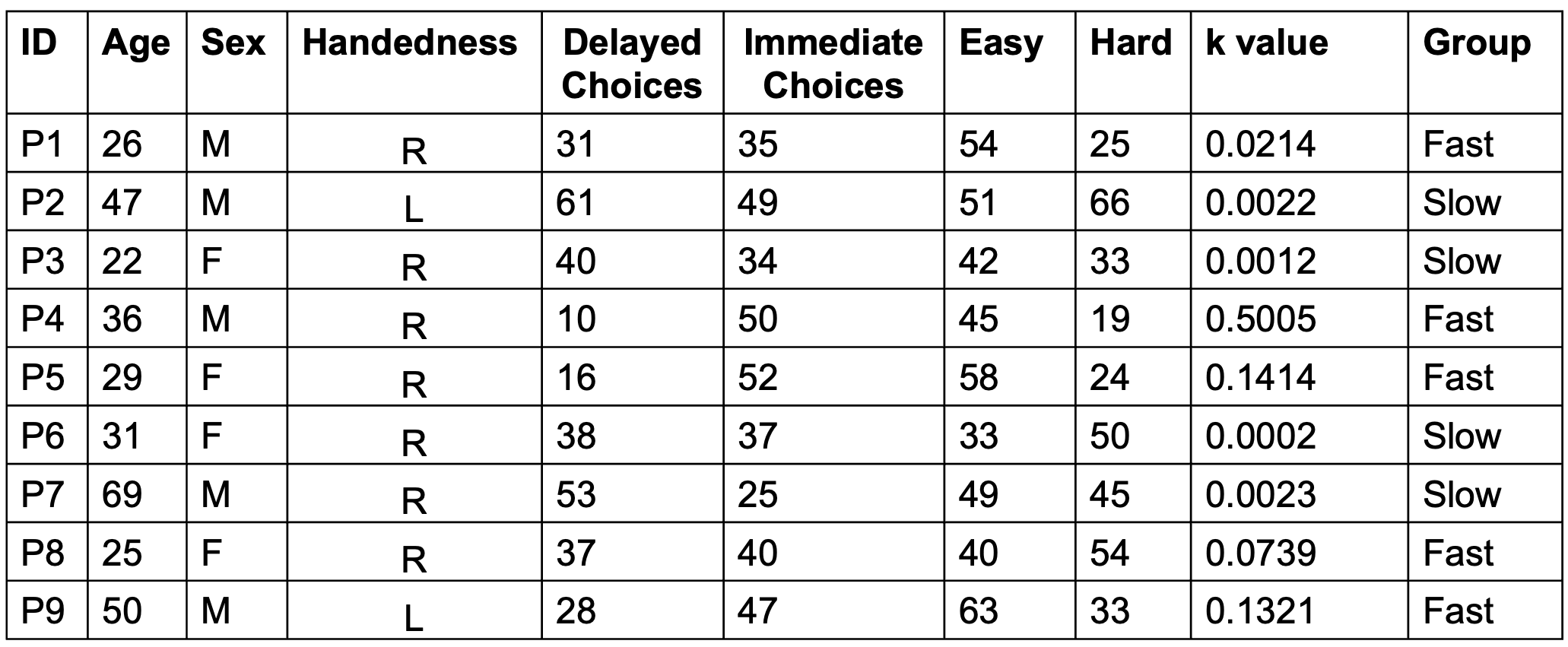


**Supplementary Table 2. Participant Behaviors.** Identification number (ID), age, sex and handedness for each included participant. Corresponding number of trials where a larger delayed option was chosen over a smaller immediate option (“Delayed Choices”) and vice versa (”Immediate Choices”). ”Easy” and “Hard” correspond to number of trials where the difference in subjective value between the immediate and delayed offer was >|$1| and <= |$1| respectively. ”k” corresponds to each subject’s discounting quotient, which was used to determine whether they were identified as a “Fast” or “Slow” discounter (“Group”; Methods).


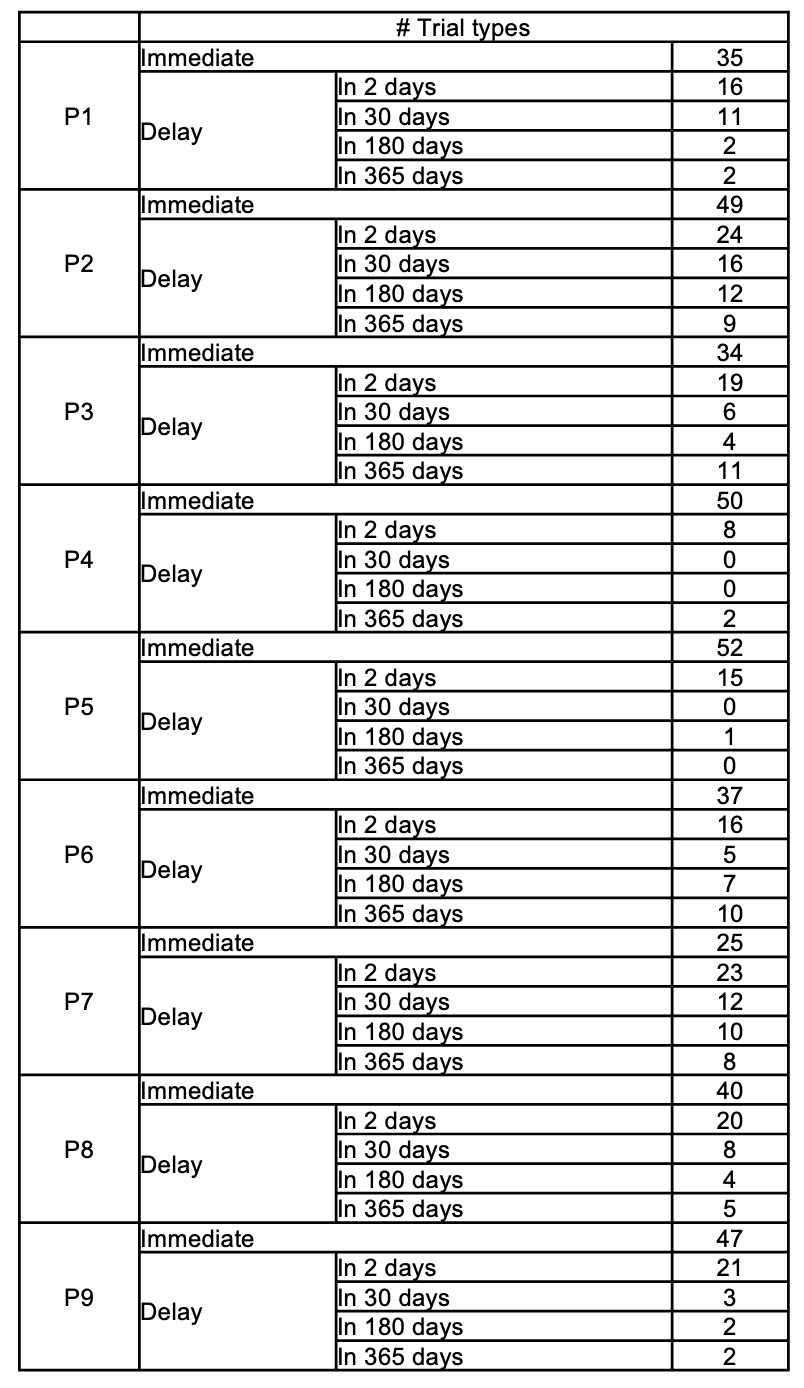


**Supplementary Table 3.** Trial Conditions by Subject. Number of immediate choice rials vs. number of delayed choice trials. During delayed choice trials, $10 to be delivered at the specified length of time (‘Delay’) over an amount less than $10 that was immediately available.
